# Supplementary material for: Sir4 Deficiency Reverses Cell Senescence by Sub-Telomere Recombination
Source: Cells. 2021 Apr 1;10(4):778. doi: 10.3390/cells10040778 (PMC8066019; doi:10.3390/cells10040778)
Supplement: Supplementary file 1 [file cells-10-00778-s001.pdf]

Supplementary Information for

Sir4 deficiency reverses yeast senescence by sub-telomere recombination

Jun Liu[1, *], Xiaojing Hong[1], Lihui Wang[1], Chao-Ya Liang[1], Jun-Ping Liu[1, 2, 3 *]

1. Institute of Ageing Research, School of Medicine, Hangzhou Normal University, Hangzhou, Zhejiang 310036, China
2. Department of Immunology, Faculty of Medicine, Monash University, Prahran, Victoria 3181, Australia
3. Hudson Institute of Medical Research, Clayton, Victoria 3168, Australia

*Correspondence authors:

Jun Liu, junliu262@hznu.edu.cn;

Jun-Ping Liu, Jun-ping.liu@monash.edu

Sporulation and micromanipulation

Briefly, diploid strains were first streaked onto fresh YPD plate and followed by two successive streakouts on fresh pre-sporulation YNA plate (1% yeast extract, 3% nutrient broth, 5% dextrose and 2% agar). In 5-10 ml liquid sporulation medium (1% potassium acetate, 0.005% zinc acetate and supplemented with 1×ura, 1×his and 1×leu), 5-6 single colonies were suspended and incubated at 24 ℃ with gentle shaking for 5 days and followed by incubation of another 3 days at 30 ℃ with shaking. For sporulation of diploid strains transformed with *CEN* plasmids, transformants were first streaked on fresh selective plates and followed by suspension of 5-6 single colonies into 10 ml synthetic complete medium dropout of appropriate amino acids for plasmids selection for 4 days to let most of cells to enter into stationary phase.

Three to five ml cultures were collected and washed twice by 40 ml sterile water. Appropriate amount of cells was suspended in liquid selective sporulation medium (1% potassium acetate, 0.005% zinc acetate and supplemented with appropriate amino acids dropped out for plasmids selection) for 5 days at 24 ℃ with gentle shaking. Cultures were incubated for another 3 days at 30 ℃ with gentle shaking. Cells was digested by zymolase (10 mg/ml) at 37 ℃ for 8-10 min, and followed by addition of 1ml ice-cold sterile water. Tetrad dissection was performed using a ZEISS yeast dissection microscope at room temperature on either YPD or selective plate.

Table S1. Yeast strains and plasmids used in this study

Strain name	Genotype	Source
BY4743	MATa/α his3Δ1/his3Δ1 leu2Δ0/leu2Δ0 LYS2/lys2Δ0 met15Δ0/MET15 ura3Δ0/ura3Δ0	Euroscarf
	BY4743 with YKU80/yku80Δ::natNT2 MRE11/ mre11Δ::HphMX6 SIR4/sir4Δ::HIS3MX6	This study
	BY4743 with YKU80/yku80Δ::natNT2 MRE11/ mre11Δ::HphMX6 SIR4/sir4Δ::HIS3MX6 RAD52/rad52Δ::KanMX6	This study
	BY4743 with YKU80/yku80Δ::natNT2 MRE11/ mre11Δ::HphMX6 SIR4/sir4Δ::HIS3MX6 RAD51/rad51Δ::URA3	This study
	BY4743 with YKU80/yku80Δ::natNT2 MRE11/ mre11Δ::HphMX6 SIR4/sir4Δ::HIS3MX6 RAD59/rad59Δ::KanMX6	This study
	BY4743 with YKU80/yku80Δ::natNT2 MRE11/ mre11Δ::HphMX6 SIR2/sir2Δ::HIS3MX6	This study
	BY4743 with YKU80/yku80Δ::natNT2 MRE11/ mre11Δ::HphMX6 SIR3/sir3Δ::HIS3MX6	This study
	BY4743 with YKU80/yku80Δ::natNT2 SIR4/sir4Δ::HIS3MX6 RIF1/rif1Δ::URA3 RIF2/rif2Δ::LEU2 MRE11/ mre11Δ::HphMX6	This study
	BY4743 with YKU80/yku80Δ::natNT2 SIR4/sir4Δ::HIS3MX6 RIF1/rif1Δ::URA3 RIF2/rif2Δ::LEU2 MRE11/ mre11Δ::HphMX6 (pRS315-yku80-135i)	This study
	BY4743 YKU80/yku80Δ::natNT2 MRE11/mre11Δ::HphMX6 SIR4/sir4Δ::HIS3MX6 SIZ2/siz2Δ::KanMX6	This study
	BY4743 YKU80/yku80Δ::natNT2 MRE11/mre11Δ::HphMX6 SIR4/sir4Δ::HIS3MX6 mps3Δ::KanMX6/mps3Δ75-150::LEU2	This study
	BY4743 YKU80/yku80Δ::natNT2 MRE11/mre11Δ::HphMX6 SIR3/sir3Δ::HIS3MX6 SIR4/sir4Δ::KanMX6	This study
	BY4743 YKU80/yku80Δ::natNT2 MRE11/mre11Δ::HphMX6 SIR4/sir4Δ::HIS3MX6	This study

	CTR9/ctr9Δ::KanMX6	
	BY4743 YKU80/yku80Δ::natNT2 MRE11/mre11Δ::HphMX6 SIR4/sir4Δ::HIS3MX6 PAF1/paf1Δ::KanMX6	This study
	**Plasmids**	**Source**
	pRS315-yku80-135i	(1)
	pFA6a-HIS3MX6	(2)
	pFA6a-KanMX6	(2)
	pFA6a-8Gly-13Myc-HphMX6	(3)
	pYM-N15	Euroscarf
	pRS306-rad51Δ cassette containing plasmid	(4)
	pRS306-rif1Δ cassette containing plasmid	(4)
	pUC19-mps3Δ75-150 (mps3Δ75-150::LEU2 integrating construct) linearized by *BamH*I digestion	This study

Table S2. Combination of primers and templates to amplify gene-specific KO cassettes

KO gene	Template	Forward primer	Reverse primer
SIR2	pFA6a-KanMX6	ATCGCTTCGGTAGACACATTCAAACCATTTTTCCCTCATCGGCACATTAAAGCTGGATGCGGATCCCCGGGTTAATTAA	ATACTATGTAAATTGATATTAATTTGGCACTTTTAAATTATTAAATTGCCTTCTACTTAGAATTCGAGCTCGTTTAAAC
SIR3	pFA6a-KanMX6	CCTTCCTTACAGGGGTTTAAGAAAGTTGTTTTGTTCTAACAATTGGATTAGCTAAAATGCGGATCCCCGGGTTAATTAA	AGACTGCATGTGTACATAGGCATATCTATGGCGGAAGTGAAAATGAATGTTGGTGGTCAGAATTCGAGCTCGTTTAAAC
SIR4	pFA6a-His3MX6	TAAAAAAAAAAAGGAAGCTTCAACCCACAATACCAAAAAAGCGAAGAAAACAGCCAATGCGGATCCCCGGGTTAATTAA	CGACAAAGAAAAACAGGGTACACTTCGTTACTGGTCTTTTGTAGAATGATAAAAAGTCAGAATTCGAGCTCGTTTAAAC
RIF2	pFA6a-KanMX6	TACGAATATAGATATAAATACGAACGTGGTTAGTATATAGAGACACGGATCCCCGGGTTAATTAA	CCATCTCTTTGTATTGTTCGAACTCTTTCAAAAGACCTTGGTAATGAATTCGAGCTCGTTTAAAC
YKU80	pYM-N15	AGAGTGCAGGACATATGCACAAATAATATATCTCACACCATAATACGTACGCTGCAGGTCGAC	TAACTGTGGTGACGAAAACATAACTCAAAGGATGTTAGACCTTTTCATCGATGAATTCTCTGTCG
MRE11	pFA6a-8G-13Myc-KanMX6	GAGAATGCAGACAATTGACGCAAGTTGTACCTGCTCAGATCCGATAAAACTCGACTATGTAACAGGGTAATATagatct	GAAGGCAAGCCCTTGGTTATAAATAGGATATAATATAATATAGGGATCAAGTACAACTAGAATTCGAGCTCGTTTAAAC
RAD52	pFA6a-KanMX6	AAACAAGGAGGTTGCCAAGAACTGCTGAAGGTTCTGGTGGCTTTGGTGTGTTGTTGATGCGGATCCCCGGGTTAATTAA	TTTTGGAGTAATAAATAATGATGCAAATTTTTTATTTGTTTCGGCCAGGAAGCGTTTCAGAATTCGAGCTCGTTTAAAC
RAD59	pFA6a-KanMX6	CCATTAAAGGGTTACGTAGAGGAGAAGAGCATATTTCAGGATAAACAGACAAAATAATGCGGATCCCCGGGTTAATTAA	CTTTTTATCAAGCAAAATAAATTTGCTACTTGTGCCCTTTTTCTTTCTTTTTTTTTTAGAATTCGAGCTCGTTTAAAC
PAF1	pFA6a-Kan	GACAGAAATGTATTCAGTACAATAGAACA	ACAAATGTAAAAAGAACTACAGGTTTA

	MX6	GTGCTCATAATAGTATAAAGGGTCACAATGCGGATCCCCGGGTTAATTAA	AAATCAATCTCCCTTCACTTCTCAATATTCTAGAATTCGAGCTCGTTTAAAC
CTR9	pFA6a-Kan MX6	TCTGTGCAAAGTTCTAATTGTCTGGTCCATTTGTGTTGAGAGCAAGAAAAAAAAACATGCGGATCCCCGGGTTAATTAA	GCAACAGTATAATATTAAGTTTCTTTAAAAGTCTTGATTCTAACCCTCGCCTCTTCTTAGAATTCGAGCTCGTTTAAAC
SIZ2	pFA6a-Kan MX6	AGAATACCACAAACGATACACTGATAATCAAGAAACGTATAAGGGAAAAGAGCACGATGCGGATCCCCGGGTTAATTAA	CAATAGAAATAAAAATAGAATACAATCGGAAAGGAAAGAAATCAAAAGACGGTTAATCAGAATTCGAGCTCGTTTAAAC
MPS3	pFA6a-Kan MX6	ACGGCGGTTTCTCTTGTTTTCAGTTGGTAGTAACTTTTATCCTGGAAGTGCTGGAAATGCGGATCCCCGGGTTAATTAA	ATCTGCGCGATTTTCTGGGGGCCAGGGGGTTAGAACGTTTAATTTTTTATTGTCGTTTAGAATTCGAGCTCGTTTAAAC

Table S3. Primers used in this study

Primer name	Sequence
rad59-A-F	CAGACAGTTGCAAATCTAAACGACT
RAD51-A-F	ATTGGCCTTTCTACTATGCCATAAA
sir3-5UTR-F	GCAATGACTGATACACAAAGAAATG
sir4-5UTR-F	ATATTTTTATCGTTGAGAACGAACG
sir2-5UTR-F	CTTTTCCAAGCTACATCTAGCACTC
mre11-orf-f	GTGAATAAGCCTTCCAAGAAGTCAC
mre11-orf-r	TCTTCAACTTGTTCAATAAGATACTTAGACG
rad51-orf-f	TGCTGAAGCGGTAGCATATGC
rad51-orf-r	CCCTTTTTGAAACCTAATCGCG
Rad51-5UTR-F	CCAATCTAGTTTAGCTATCCTGCAA
T7	TAATACGACTCACTATAGGG
rad59-orf-f	TCGAGCATATCGTATGATTCGACT
rad59-orf-r	GTCGCTGACAATAAGTTGTAATTGG
rad52-orf-f	GGTAATGCTCTAGGAAATTGTCTTTACG
rad52-orf-r	CGCTGGAATATGCTTGGACG
Hygro-R	TATCCACGCCCTCCTACATC
KanMX-R	CTGCAGCGAGGAGCCGTAAT
natGPD-R	ATAAGAGCGACCTCATGCTATACCT
mre11-5UTR-F	GTTCACAAGCAAGCCTGTAAATAAT
mre11-3UTR-R	ATTCCTTGCTATACGAACAAAAGAG
YKU80-ORF-F	TCAACACAAACATCGAATCCT
YKU80-ORF-R	TGCTCAATTAGAACGGGACA
SIR3-ORF-F	ACGGCCGAGAGAATTTTGTA
SIR3-ORF-R	TTTGCTTTCGGGACATTTG
SIR2-ORF-F	CAAGGGGCCAATCATAATCA
SIR2-ORF-R	TAACAGTACGGGCATAGTGGA
SIR4-ORF-F	TCAAACGTACTCAAGGCTCCT
SIR4-ORF-R	ACACGGTTGGAATTTGGGAT
PAF1-ORF-F	GAAAAGGAAAAGGCGCTTG

PAF1-ORF-R	TTCGATCTTTGAACGTAGGG
CTR9-ORF-F	TCCAGGAATCCAAAGGAACA
CTR9-ORF-R	TCCAGGGAATCCTTGATTTG
SIZ2-ORF-F	TATGAAACTGTGGGCCATGA
SIZ2-ORF-R	TTCGGAAATCTTCAACTGGTC
MPS3-ORF-F	AGAAGACAGCGATTCCGACA
MPS3-ORF-R	CCAGTTGATCTTGCCATTGA
mps3-leu2-f	CGCTTCTAACGTCCCATCAT
mps3-leu2-r	CCATTTAGGACCACCCACAG
PAF1-5UTR-F	ATATCGAAGTTGACCATACGAAAAG
CTR9-5UTR-F	TTCTGCCAGCAATAACTTCCT
SIZ2-5UTR-F	TGTTCAACAAAATGTGATAAAGGAA
MPS3-5UTR-F	GGGACGCGATTGATGTTTAC
mps3Δ75-150-f1	CGACGATGATGATCCGTACA
mps3Δ75-150-r1	TGAGGGAGGTGTGTATCCAA
mps3-5UTR-f2	GGAGGTAAAATAGGGGCATTG
mps3Δ75-150-r2	GTAGCTGTCGGAATCGCTGT

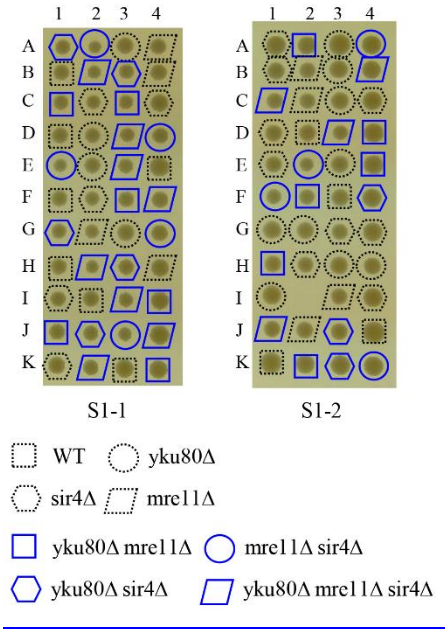

Fig. S1 Tetrad dissection of diploid heterozygous for yku80Δ, mre11Δ and sir4Δ

The diploid heterozygous for yku80Δ::natNT2, mre11Δ::HphMX6 and sir4Δ::HIS3MX6 was subjected to sporulation and tetrad dissection on YPD plate (plate S1-1 and S1-2) to obtain indicated spores with 8 different genotypes. The genotype of each spore was determined by streaking each individual spore on –His, CloNAT and Hygromycin B plates, respectively.

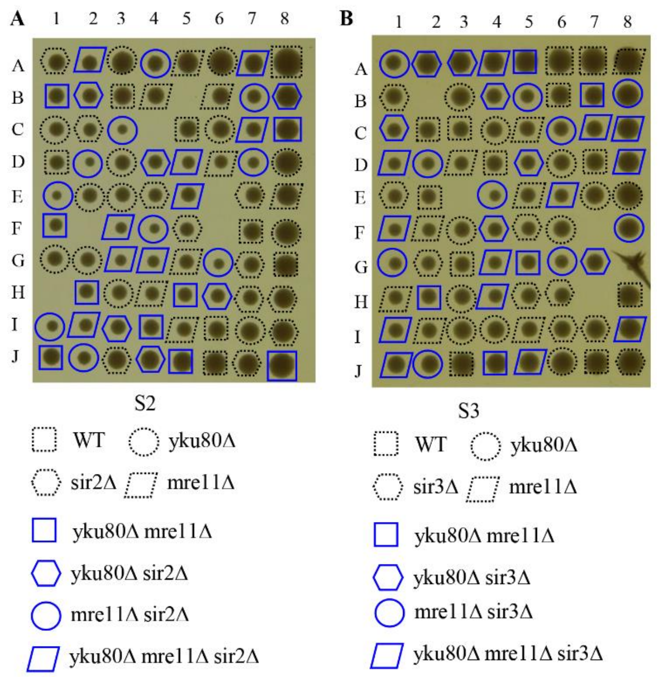

Fig. S2 Tetrad dissection of two diploid strains heterozygous for yku80Δ mre11Δ sir2Δ and yku80Δ mre11Δ sir3Δ

(A) The diploid heterozygous for yku80Δ::natNT2, mre11Δ::HphMX6 and sir2Δ::HIS3MX6 was subjected to sporulation and tetrad dissection on YPD plate (plate S2) to obtain indicated spores with 8 different genotypes. The genotype of each spore was determined by streaking each individual spore on –His, CloNAT and Hygromycin B plates, respectively. (B) The diploid heterozygous for yku80Δ::natNT2, mre11Δ::HphMX6 and sir3Δ::HIS3MX6 was subjected to sporulation and tetrad dissection on YPD plate (plate S3) to obtain indicated spores with 8 different genotypes. The genotype of each spore was determined by streaking each individual spore on –His, CloNAT and Hygromycin B plates, respectively.

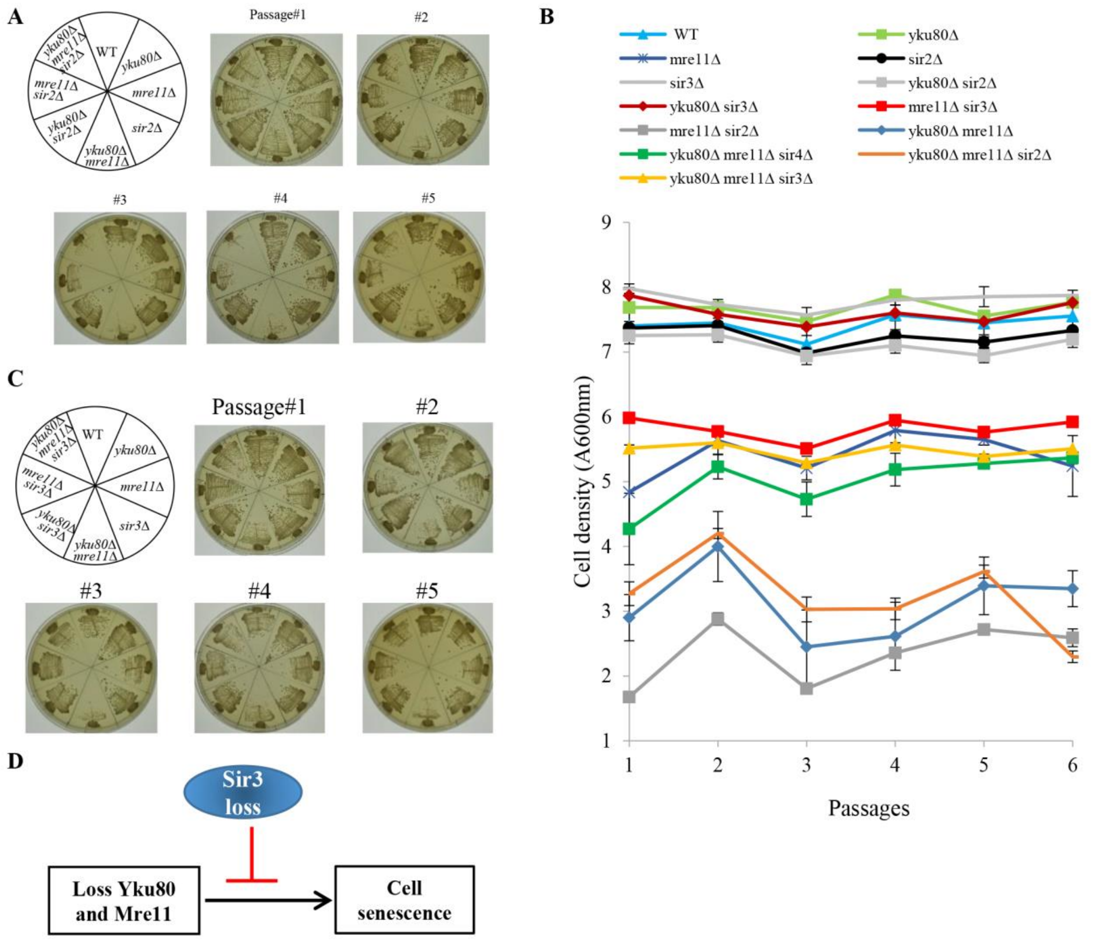

Fig. S3: Effects of *SIR3* or *SIR2* deletion on cell senescence of *yku80*Δ *mre11*Δ cells. (A) Spore WT, *yku80*Δ, *mre11*Δ, *sir2*Δ, *yku80*Δ *mre11*Δ, *yku80*Δ *sir2*Δ, *mre11*Δ *sir2*Δ and *yku80*Δ *mre11*Δ *sir2*Δ cells were successively streaked on YPD plates every 2 days for six times. Images of photographed colony growth on solid media are from one of typical 5 experiments. (B) Spore WT, *yku80*Δ, *mre11*Δ, *sir2*Δ, *sir3*Δ, *yku80*Δ *sir2*Δ, *yku80*Δ *sir3*Δ, *mre11*Δ *sir3*Δ, *mre11*Δ *sir2*Δ, *yku80*Δ *mre11*Δ, *yku80*Δ *mre11*Δ *sir4*Δ, *yku80*Δ *mre11*Δ *sir2*Δ and *yku80*Δ *mre11*Δ *sir3*Δ cells were cultured in liquid YPD medium with initial concentration of $A600_{nm}$=0.01 for around 20 hrs and followed by $A600_{nm}$ measurement, and re-dilution was made every 20 hrs. Results are mean ± SEM (n=5). (C) Spore cells WT, *yku80*Δ, *mre11*Δ, *sir3*Δ, *yku80*Δ *mre11*Δ, *yku80*Δ *sir3*Δ, *mre11*Δ *sir3*Δ and *yku80*Δ *mre11*Δ *sir3*Δ were successively streaked on YPD plates every 2 days for six times. Images of photographed colony growth on solid media are from one of 5 typical experiments. (D) Graphic summary of the effect of Sir3 on cell senescence.

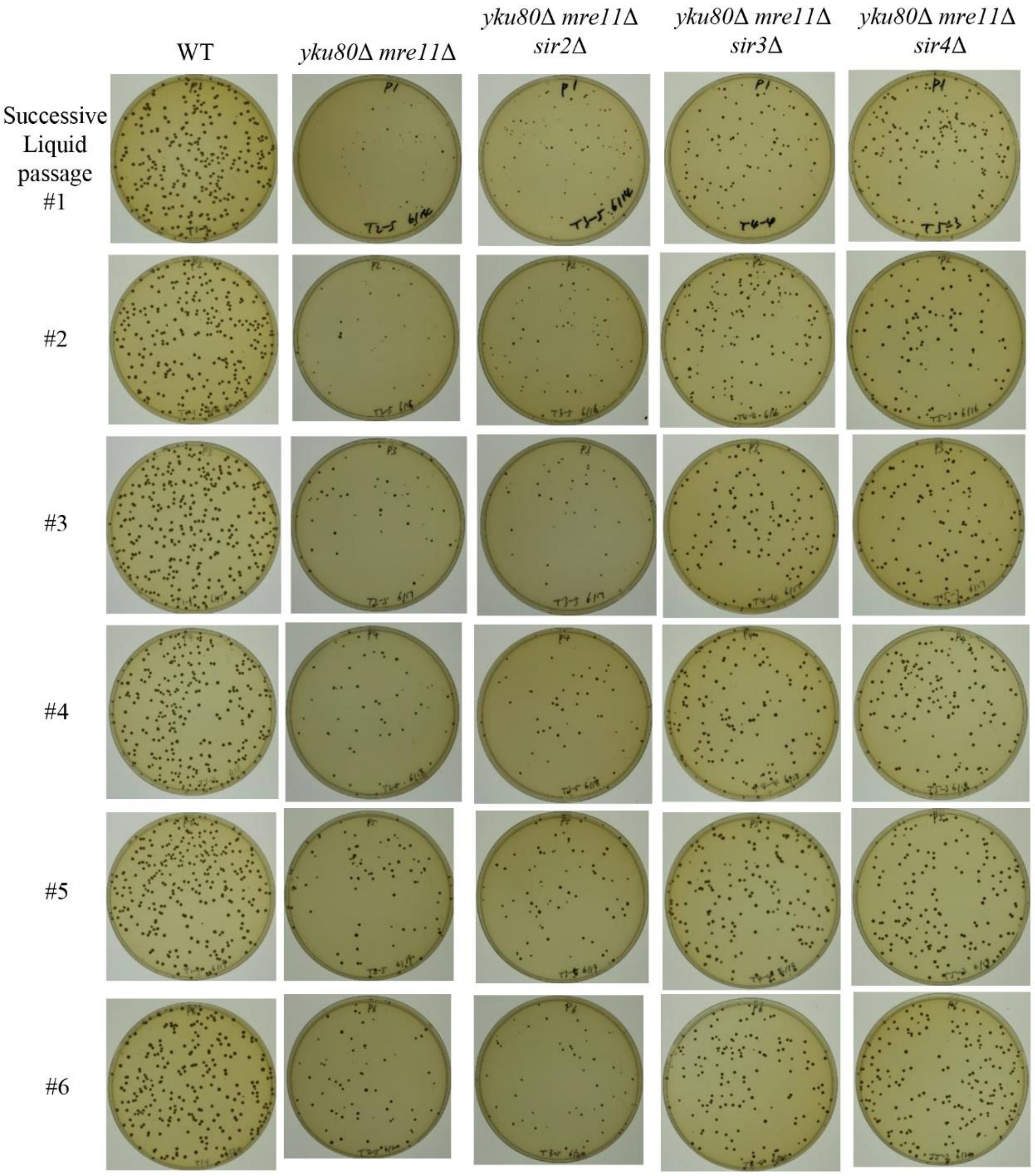

Fig. S4 Effects of SIR2, *SIR3* or SIR4 deletion on growth of *yku80*Δ *mre11*Δ cells. Spore cells of WT, *yku80*Δ *mre11*Δ, *yku80*Δ *mre11*Δ *sir2*Δ, *yku80*Δ *mre11*Δ *sir3*Δ, *yku80*Δ *mre11*Δ *sir4*Δ were successively cultured in liquid YPD medium with initial concentration of $A600_{nm}$=0.01 for around 20 hrs and followed by $A600_{nm}$ measurement and re-dilution was made every 20 hrs. For each passage, cultures were equal volume diluted with the same dilution factor and plated onto fresh YPD plates, incubated 3days before taking photos.

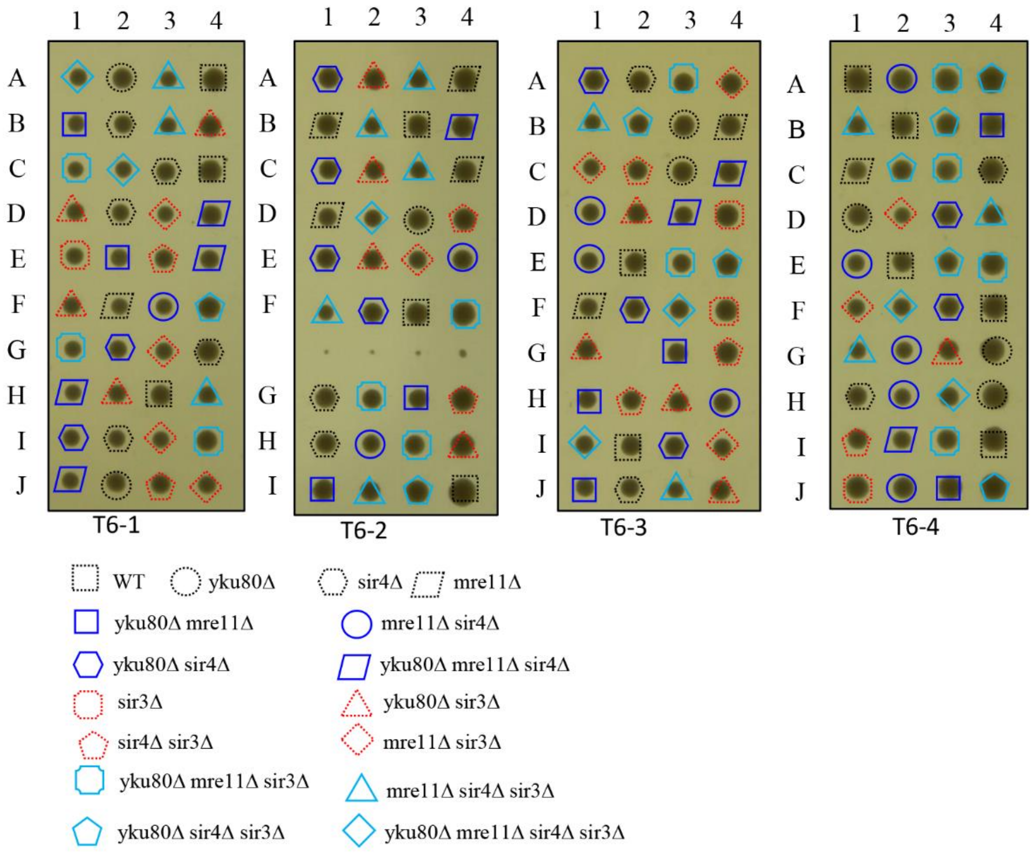

Fig. S5. Tetrad dissection diploid heterozygous for yku80Δ mre11Δ sir3Δ sir4Δ

Diploid strain BY4743 YKU80/yku80Δ::natNT2 MRE11/mre11Δ::HphMX6 SIR3/ sir3Δ::HIS3MX6 SIR4/sir4Δ::KanMX6 were sprorulated and tetrad dissection was done on YPD. Genotype of each spore was determined by streaking every spore onto selective plates including ClonNAT, G418, Hygromycin B and –His plates.

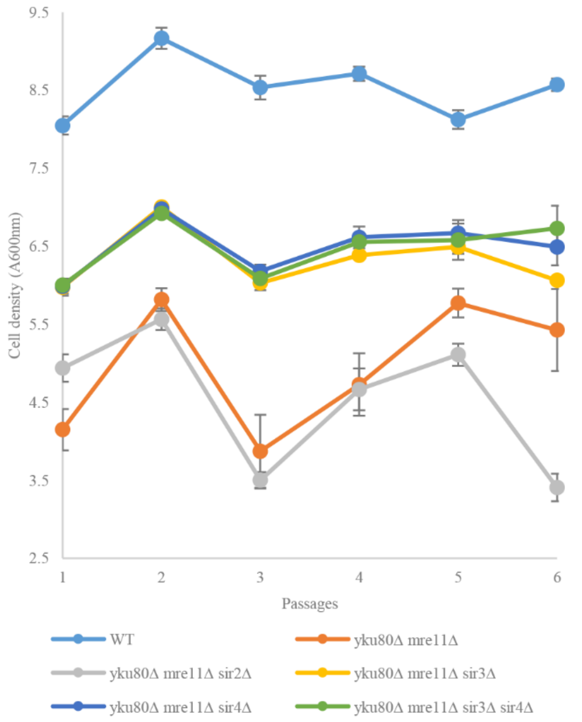

Fig. S6 Effects of *SIR3* and SIR4 deletion on cell senescence of *yku80*Δ *mre11*Δ cells. (A) Spore cells of WT, *yku80*Δ *mre11*Δ, *yku80*Δ *mre11*Δ *sir2*Δ, *yku80*Δ *mre11*Δ *sir3*Δ, *yku80*Δ *mre11*Δ *sir4*Δ and *yku80*Δ *mre11*Δ *sir3*Δ *sir4*Δ were successively cultured in liquid YPD medium with initial concentration of $A600_{nm}$=0.01 for around 20 hrs and followed by $A600_{nm}$ measurement, and re-dilution was made every 20 hrs. Results are mean ±SEM (n=3).

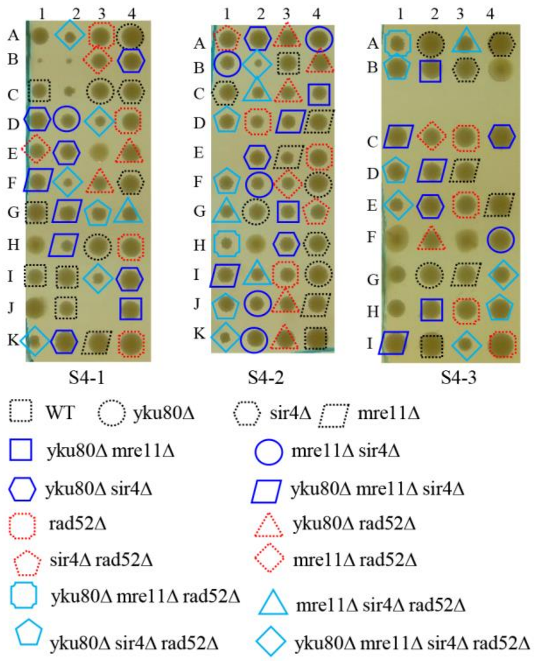

Fig. S7 Tetrad dissection of diploid heterozygous for yku80∆, mre11∆ sir4∆ and rad52∆

(A) The diploid heterozygous for yku80∆::natNT2, mre11∆::HphMX6, sir4∆::HIS3MX6 and rad52∆::KanMX6 was subjected to sporulation and tetrad dissection on YPD plate (plate S4-1, S4-2 and S4-3) to obtain indicated spores with 16 different genotypes. The genotype of each spore was determined by streaking each individual spore on –His, CloNAT, G418 and Hygromycin B plates, respectively.

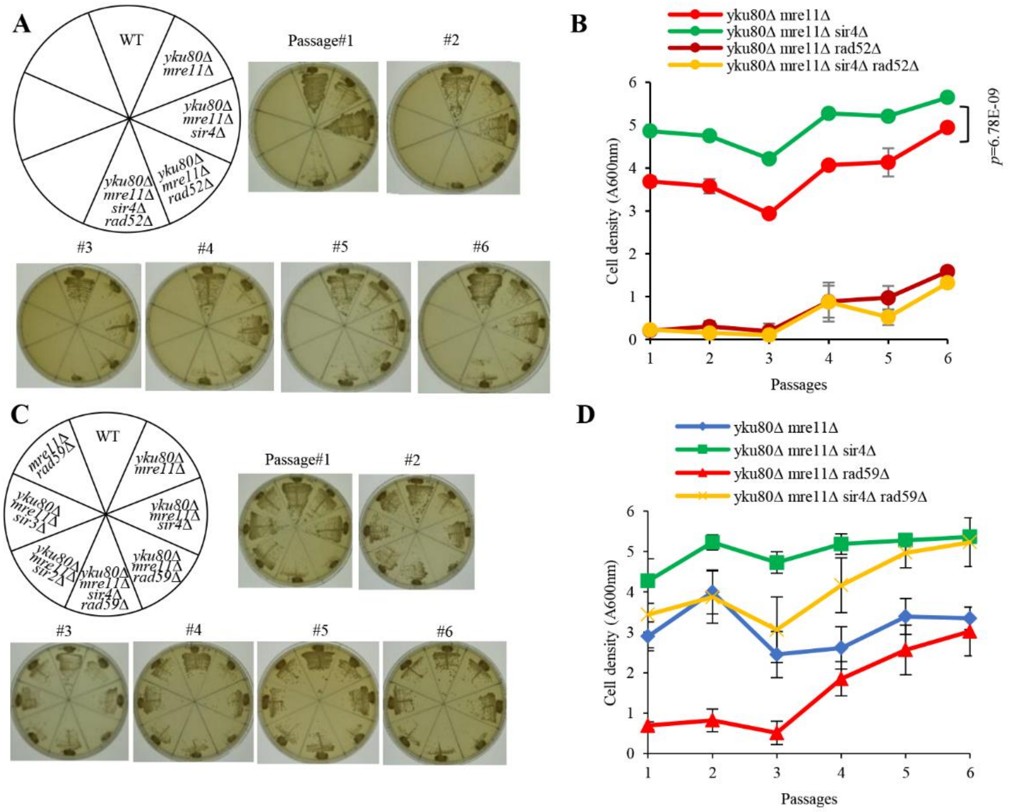

Fig. S8 Senescence analysis for yku80Δ mre11Δ and yku80Δ mre11Δ sir4Δ cell with/without rad52Δ or rad59Δ

Spore cells with indicated genotypes were successively streaked onto YPD plates every 48hrs or passaged in YPD liquid medium successively every 20hrs.

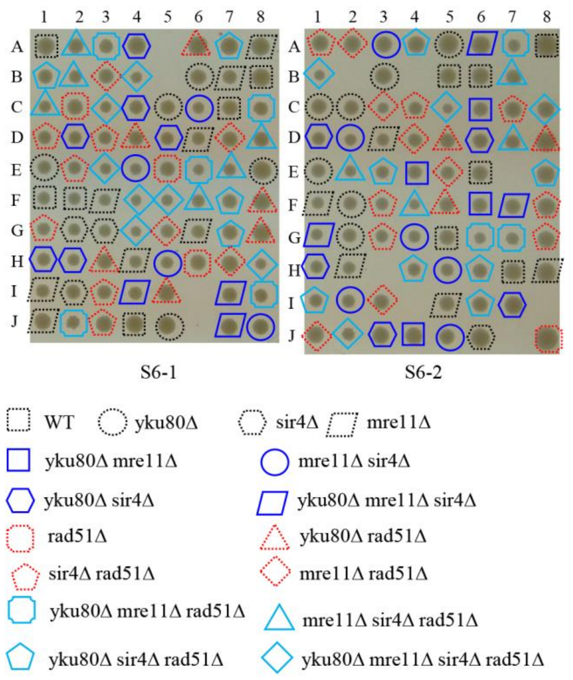

Fig. S9 Tetrad dissection of diploid heterozygous for yku80Δ, mre11Δ sir4Δ and rad51Δ

(A) The diploid heterozygous for yku80Δ::natNT2, mre11Δ::HphMX6, sir4Δ::HIS3MX6 and rad51Δ::URA3 was subjected to sporulation and tetrad dissection on YPD plate (plate S6-1 and S6-2) to obtain indicated spores with 16 different genotypes. The genotype of each spore was determined by streaking each individual spore on –His, CloNAT, –Ura and Hygromycin B plates, respectively. (B) Spores with indicated genotypes were successively streaked onto YPD plates and photographed every 48 hrs.

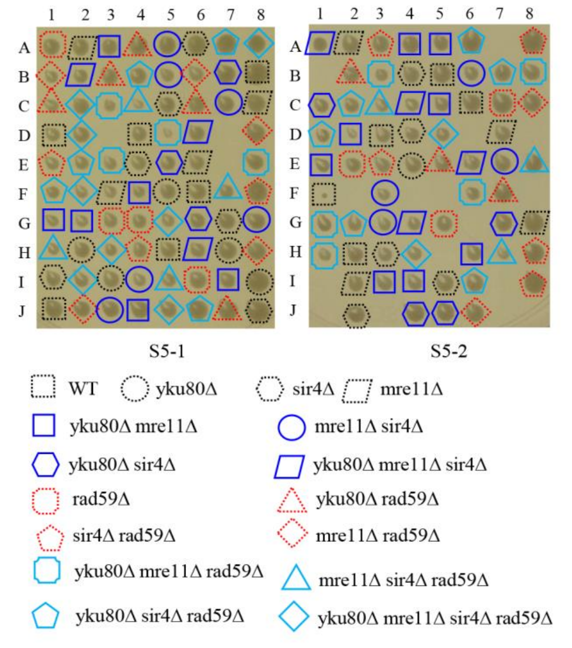

Fig. S10 Tetrad dissection of diploid heterozygous for yku80Δ, mre11Δ sir4Δ and rad59Δ

The diploid heterozygous for yku80Δ::natNT2, mre11Δ::HphMX6, sir4Δ::HIS3MX6 and rad59Δ::KanMX6 was subjected to sporulation and tetrad dissection on YPD plate (plate S5-1 and S5-2) to obtain indicated spores with 16 different genotypes. The genotype of each spore was determined by streaking each individual spore on –His, CloNAT, G418 and Hygromycin B plates, respectively.

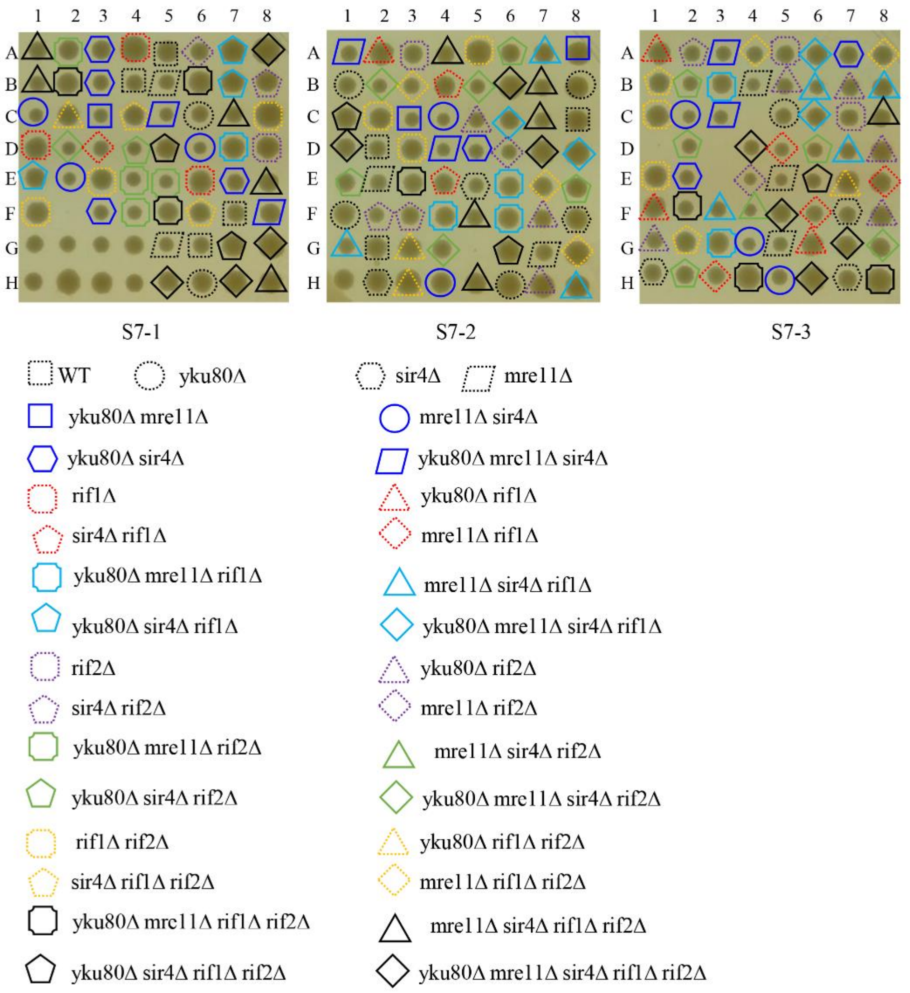

Fig. S11 Tetrad dissection of diploid heterozygous for rif1Δ rif2Δ mre11Δ yku80Δ and sir4Δ

The diploid heterozygous for yku80Δ::natNT2, rif2Δ::KanMX6, sir4Δ::HIS3MX6, rif1Δ::URA3, mre11Δ::HphMX6 was subjected to sporulation and tetrad dissection on YPD plate (plate S7-1, S7-2 and S7-3) to obtain indicated spores with 32 different genotypes. The genotype of each spore was determined by streaking each individual spore on –Ura, –His, CloNAT, G418 and Hygromycin B plates, respectively.

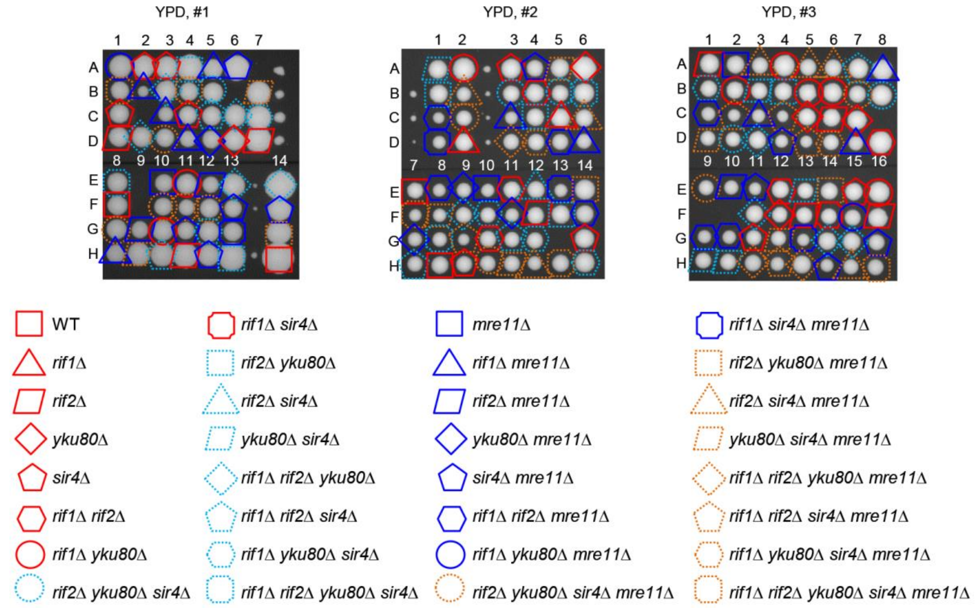

spore genotype	Spore#1	Spore#2
WT	#1-8F	#1-14H
rif1Δ	#2-2D	#2-5C
rif2Δ	#1-1D	#1-11H
yku80Δ	#1-6D	#2-6A
sir4Δ	#1-1C	#1-4C
rif1Δ rif2Δ	#2-4B	#2-10G
rif1Δ *yku80Δ*	#1-11E	#1-10G
rif1Δ sir4Δ	#3-14F	
rif2Δ *yku80Δ*	#1-4A	#1-13H
rif2Δ sir4Δ	#2-12E	
yku80Δ sir4Δ	#2-1A	#3-3B
rif1Δ rif2Δ *yku80Δ*	#1-2D	#1-3B
rif1Δ rif2Δ sir4Δ	#1-5C	#1-10H
rif1Δ *yku80Δ* sir4Δ	#1-12G	#2-10F
rif2Δ *yku80Δ* sir4Δ	#1-5B	#1-7C
rif1Δ rif2Δ *yku80Δ* sir4Δ	#1-8E	#2-6B

spore genotype	Spore#1	Spore#2
mre11Δ	#1-10E	#2-10E
rif1Δ mre11Δ	#1-2B	#1-3C
rif2Δ mre11Δ	#1-9G	#1-12E
yku80Δ mre11Δ	#1-5D	#2-7G
sir4Δ mre11Δ	#1-13F	#1-14F
rif1Δ rif2Δ mre11Δ	#2-1C	#2-5D
rif1Δ *yku80Δ* mre11Δ	#1-1A	#3-15F
rif1Δ sir4Δ mre11Δ	#1-13G	#2-1D
rif2Δ *yku80Δ* mre11Δ	#2-14E	#3-14E
rif2Δ sir4Δ mre11Δ	#2-2B	#2-6C
yku80Δ sir4Δ mre11Δ	#1-7B	#1-9H
rif1Δ rif2Δ *yku80Δ* mre11Δ	#2-3D	#3-11H
rif1Δ rif2Δ sir4Δ mre11Δ	#2-9G	#3-2C
rif1Δ *yku80Δ* sir4Δ mre11Δ	#2-4D	#3-6D
rif2Δ *yku80Δ* sir4Δ mre11Δ	#1-3D	#1-10F
rif1Δ rif2Δ *yku80Δ* sir4Δ mre11Δ	#1-1B	#1-14G

Fig. S12 Tetrad dissection of diploid heterozygous for rif1Δ rif2Δ mre11Δ yku80Δ and sir4Δ

The diploid heterozygous for yku80Δ::natNT2, rif2Δ::KanMX6, sir4Δ::HIS3MX6, rif1Δ::URA3, mre11Δ::HphMX6 was subjected to sporulation and tetrad dissection on YPD plate (plate#1-#3) to obtain indicated spores with 32 different genotypes. The genotype of each spore was determined by streaking each individual spore on –Ura, –His, CloNAT, G418 and Hygromycin B plates, respectively.

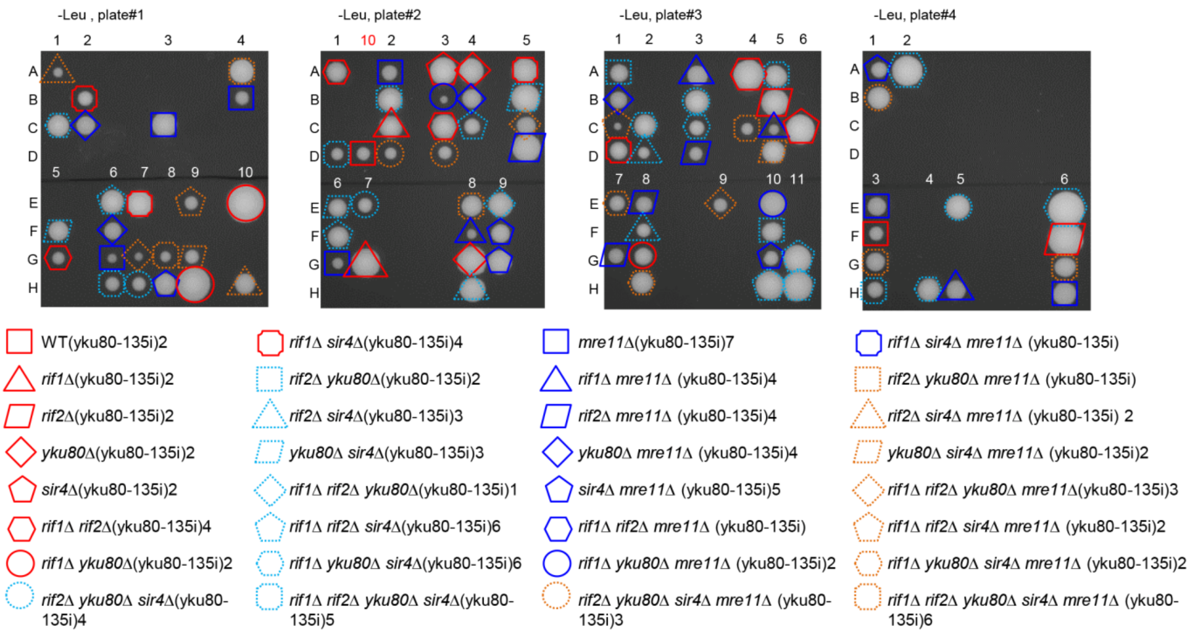

spore genotype	Spore#1	Spore#2
WT (yku80-135i)	#2-10D	#4-3F
rif1Δ (yku80-135i)	#2-2C	#2-7G
rif2Δ (yku80-135i)	#3-5B	#4-6F
yku80Δ (yku80-135i)	#2-4A	#2-8G
sir4Δ (yku80-135i)	#2-3A	#3-6C
rif1Δ rif2Δ (yku80-135i)	#1-5G	#2-3C
rif1Δ *yku80Δ* (yku80-135i)	#1-9H	#3-8G
rif1Δ sir4Δ (yku80-135i)	#2-5A	#3-1D
rif2Δ *yku80Δ* (yku80-135i)	#3-1A	#3-10F
rif2Δ sir4Δ (yku80-135i)	#2-8H	#3-8F
yku80Δ sir4Δ (yku80-135i)	#2-5B	#2-6E
rif1Δ rif2Δ *yku80Δ* (yku80-135i)	#2-9E	
rif1Δ rif2Δ sir4Δ (yku80-135i)	#1-6E	#2-6F
rif1Δ *yku80Δ* sir4Δ (yku80-135i)	#3-3C	#1-1C
rif2Δ *yku80Δ* sir4Δ (yku80-135i)	#1-7H	#3-3B
rif1Δ rif2Δ *yku80Δ* sir4Δ (yku80-135i)	#1-6H	#3-5A

spore genotype	Spore#1	Spore#2
mre11Δ (yku80-135i)	#1-6G	#2-2A
rif1Δ mre11Δ (yku80-135i)	#2-8F	#3-5C
rif2Δ mre11Δ (yku80-135i)	#3-3D	#3-8E
yku80Δ mre11Δ (yku80-135i)	#1-6F	#3-1B
sir4Δ mre11Δ (yku80-135i)	#3-10G	#2-9F
rif1Δ rif2Δ mre11Δ (yku80-135i)		
rif1Δ *yku80Δ* mre11Δ (yku80-135i)	#2-3B	#3-10E
rif1Δ sir4Δ mre11Δ (yku80-135i)		
rif2Δ *yku80Δ* mre11Δ (yku80-135i)		
rif2Δ sir4Δ mre11Δ (yku80-135i)	#1-1A	#1-10H
yku80Δ sir4Δ mre11Δ (yku80-135i)	#1-9G	#3-5D
rif1Δ rif2Δ *yku80Δ* mre11Δ (yku80-135i)	#1-7G	#2-5C
rif1Δ rif2Δ sir4Δ mre11Δ (yku80-135i)	#1-9E	#3-1C
rif1Δ *yku80Δ* sir4Δ mre11Δ (yku80-135i)	#3-7E	#3-8H
rif2Δ *yku80Δ* sir4Δ mre11Δ (yku80-135i)	#2-2D	#2-3D
rif1Δ rif2Δ *yku80Δ* sir4Δ mre11Δ (yku80-135i)	#2-8E	#4-3G

Fig. S13 Tetrad dissection of diploid heterozygous for rif1Δ rif2Δ mre11Δ yku80Δ and sir4Δ carrying pRS315-yku80-135i

The diploid heterozygous for yku80Δ::natNT2, rif2Δ::KanMX6, sir4Δ::HIS3MX6, rif1Δ::URA3, mre11Δ::HphMX6 and carrying CEN plasmid pRS315-yku80-135i was subjected to sporulation and tetrad dissection on -Leu plate for plasmid selection (plate#1-#4) to obtain spores with indicated 32 different genotypes. The genotype of each spore was determined by streaking each individual spore on –Ura, –His, CloNAT, G418 and Hygromycin B plates, respectively.

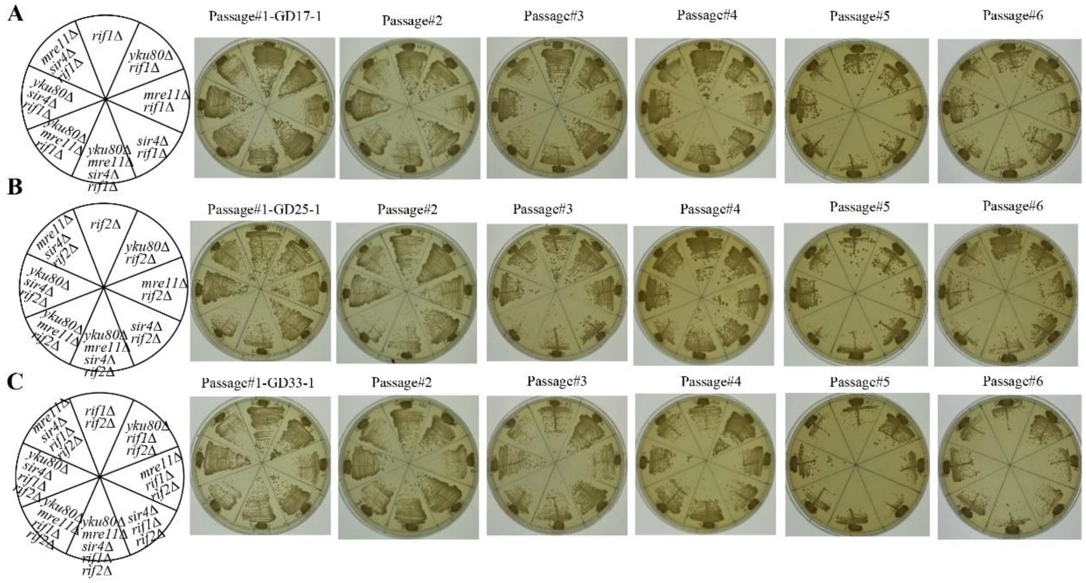

Fig. S14 Successive streakouts of spores with different genotypes

In Figure S6, the diploid heterozygous for yku80Δ::natNT2, rif2Δ::KanMX6, sir4Δ::HIS3MX6, rif1Δ::URA3, mre11Δ::HphMX6 was subjected to sporulation and tetrad dissection on YPD plate (plate S7-1, S7-2 and S7-3) to obtain indicated spores with 32 different genotypes. The genotype of each spore was determined by streaking each individual spore on –Ura, –His, CloNAT, G418 and Hygromycin B plates, respectively. Indicated spores were successively streaked on YPD plates every 48 hrs and followed by photographing and restreaking on fresh YPD plates.

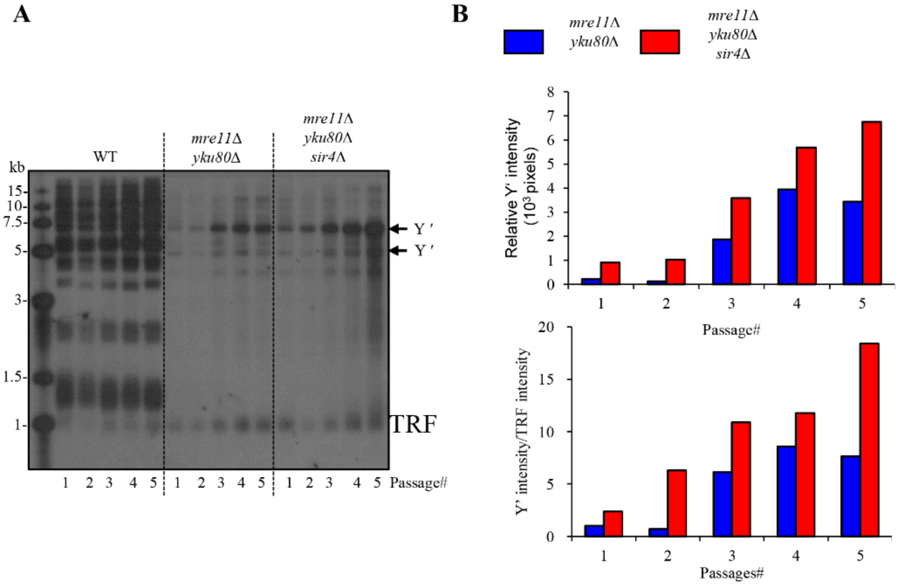

Fig. S15. Effects of Sir4 deficiency on Y' element amplification of yku80Δ mre11Δ cells

(A) WT, *yku80Δ mre11Δ*, *mre11Δ sir4Δ* and *yku80Δ mre11Δ sir4Δ* spore cells were cultured in liquid medium with initial concentration of A600=0.01 for 48hrs and followed by $A600_{nm}$ measurement, and re-dilution was made every 48 hrs. Cells were collected from each passage and subjected to total DNA extraction and telomere blot with a TG_{1-3} probe. (B) Upper panel: quantitated Y' element intensity results from (A) and lower panel: the ratio of Y' element intensity and the intensity of *XhoI* terminal restriction fragment (TRF).

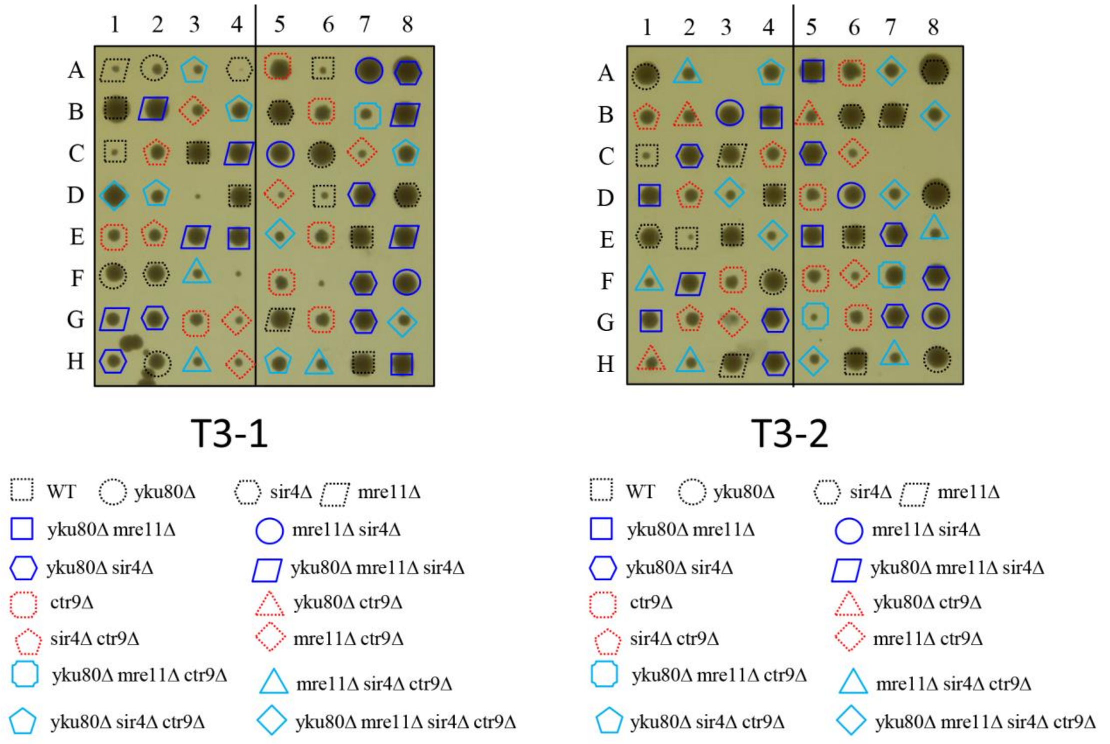

Fig. S16 Tetrad dissection of diploid strain heterozygous for yku80Δ mre11Δ sir4Δ ctr9Δ The diploid heterozygous for yku80Δ::natNT2, mre11Δ::HphMX6 and sir4Δ::HIS3MX6 ctr9Δ::KanMX6 was subjected to sporulation and tetrad dissection on YPD plate (plates T3-1 and T3-2) to obtain indicated spores with 8 different genotypes. The genotype of each spore was determined by streaking each individual spore on –His, CloNAT, G418 and Hygromycin B plates, respectively.

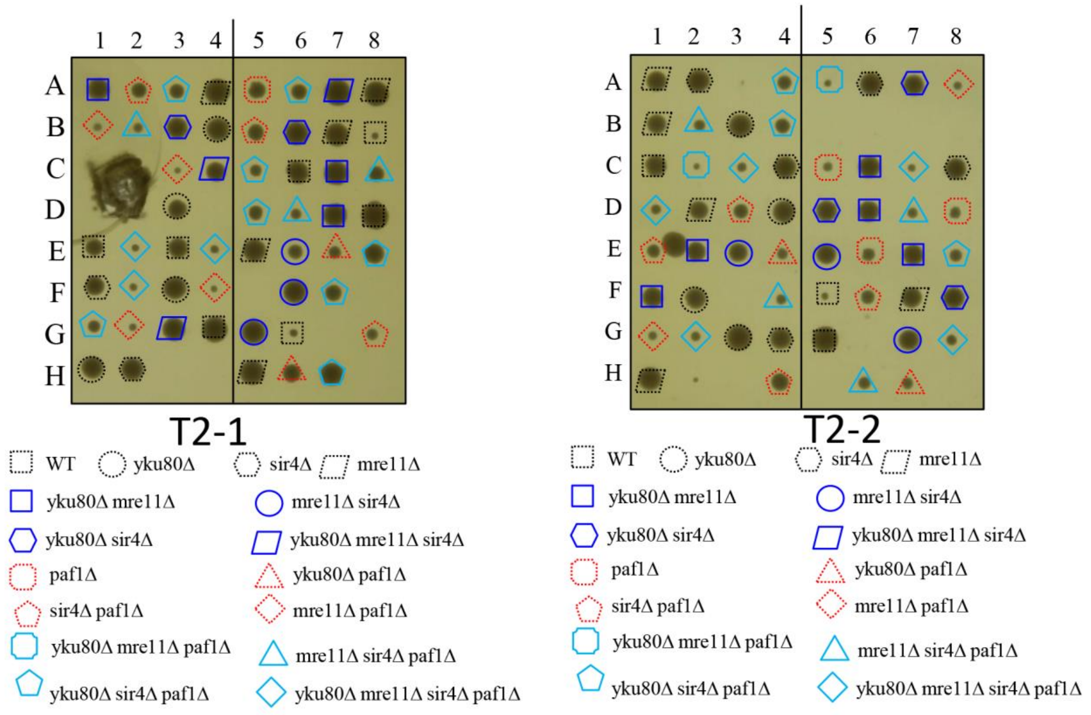

Fig. S17 Tetrad dissection of diploid strain heterozygous yku80Δ mre11Δ sir4Δ ctr9Δ
The diploid heterozygous for yku80Δ::natNT2, mre11Δ::HphMX6 and sir4Δ::HIS3MX6 ctr9Δ::KanMX6 was subjected to sporulation and tetrad dissection on YPD plate (plates T2-1 and T2-2) to obtain indicated spores with 8 different genotypes. The genotype of each spore was determined by streaking each individual spore on –His, CloNAT, G418 and Hygromycin B plates, respectively.

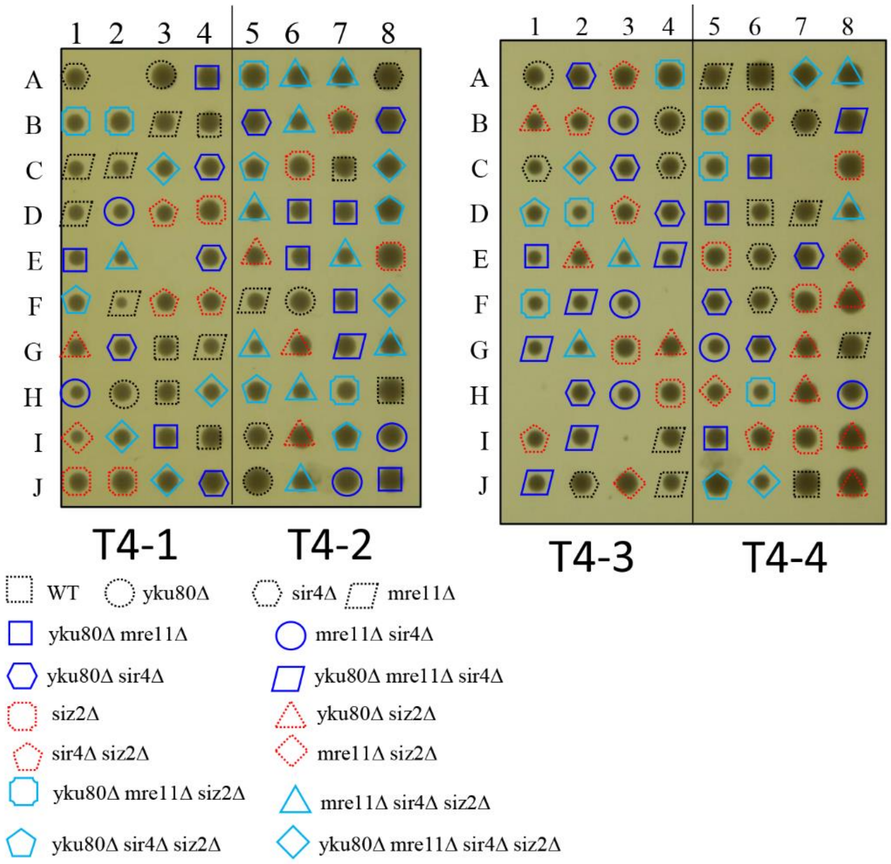

Fig. S18 BY4743 YKU80/yku80Δ::natNT2 MRE11/mre11Δ::HphMX6 SIR4/ sir4Δ::HIS3MX6 SIZ2/siz2Δ::KanMX6 tetrad dissection

The diploid heterozygous for yku80Δ::natNT2, mre11Δ::HphMX6 and sir4Δ::HIS3MX6 SIZ2/siz2Δ::KanMX6 was subjected to sporulation and tetrad dissection on YPD plate (plates T4-1, T4-2, T4-3, T4-4) to obtain indicated spores with 8 different genotypes. The genotype of each spore was determined by streaking each individual spore on –His, CloNAT, G418 and Hygromycin B plates, respectively.

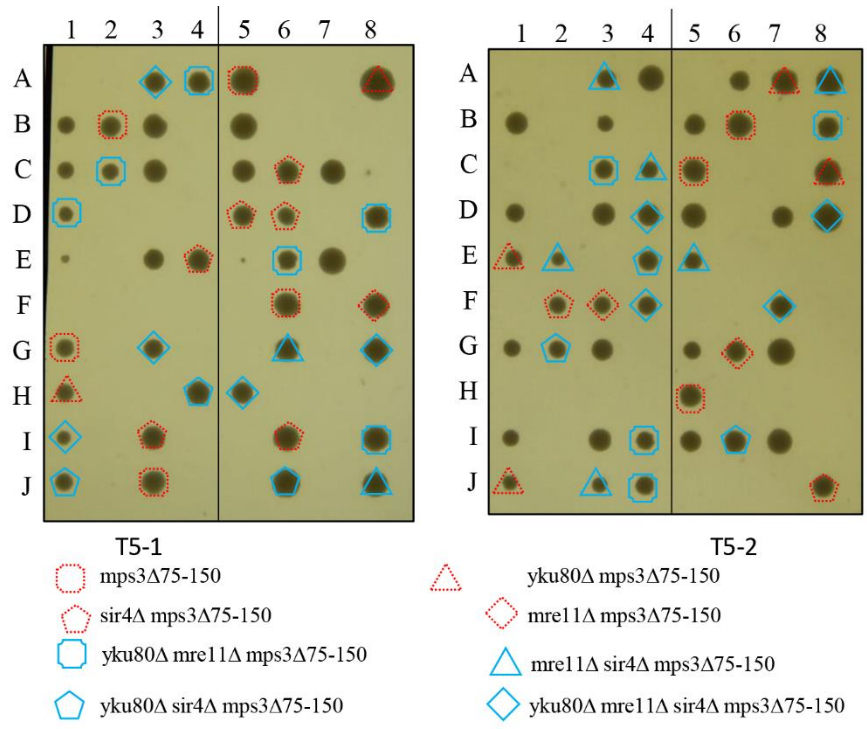

Fig. S19. Tetrad dissection of BY4743 YKU80/yku80Δ::natNT2 MRE11/mre11Δ::HphMX6 SIR4/sir4Δ::HIS3MX6 mps3Δ::KanMX6/mps3Δ75-150::LEU2

The diploid heterozygous for yku80Δ::natNT2, mre11Δ::HphMX6 and sir4Δ::HIS3MX6 mps3Δ::KanMX6/mps3Δ75-150::LEU2 was subjected to sporulation and tetrad dissection on YPD plate (plates T5-1, T5-2) to obtain indicated spores with 8 different genotypes. The genotype of each spore was determined by streaking each individual spore on –His, -Leu, CloNAT, G418 and Hygromycin B plates, respectively.

SI Reference

1. Stellwagen AE, Haimberger ZW, Veatch JR, & Gottschling DE (2003) Ku interacts with telomerase RNA to promote telomere addition at native and broken chromosome ends. *Genes & Development* 17(19):2384-2395.
2. Longtine MS*, et al.* (1998) Additional modules for versatile and economical PCR-based gene deletion and modification in Saccharomyces cerevisiae. *Yeast* 14(10):953-961.
3. Chen H*, et al.* (2018) Structural Insights into Yeast Telomerase Recruitment to Telomeres. *Cell* 172.
4. Wu Z*, et al.* (2017) Rad6-Bre1-mediated H2B ubiquitination regulates telomere replication by promoting telomere-end resection. *Nucleic Acids Research* 45(6):3308-3322.
